# A Practical Treatise on Diseases of the Eye

**Published:** 1855-11

**Authors:** 


ART. VIII.—*A Practical Treatise on Diseases of the Eye.* By WILLIAM MACKENZIE, M. D., Surgeon Oculist in Ordinary to Her Majesty; Lecturer on the Eye in the University of Glasgow, and one of the Surgeons to the Glasgow Eye Infirmary. To which is prefixed, an Anatomical Introduction explanatory of a Horizontal Section of the Human Eyeball. By THOMAS WHARTON JONES, F. R. S., Professor of Opthalmic Medicine and Surgery in University College, London, and Opthalmic Surgeon to the Hospital. With one hundred and seventy-five illustrations. From the fourth revised and enlarged London Edition. With Notes and Additions, by ADDINELL HEWSON, A. M., M. D., one of the Surgeons to Wills Hospital for Diseases of the Eye; Lecturer on Surgery in the Philadelphia Association for Medical Instruction, etc., etc. Philadelphia: Blanchard & Lea. 1855.

So comprehensive a title takes the wind out of the sails of the reviewer, were it indeed possible for us to give any sufficient account of the contents of this large octavo, which is fully equal in size, and we presume in merit, to that by Sir Wm. Lawrence, recently republished.

It is said of this work of Dr. Mackenzie's, that "it indisputably holds the first place abroad among the valuable systematic treatises published there on diseases of the eye, and forms, in respect of learning and research, an encyclopedia unequalled in extent by any othor work of the kind, either English or foreign."

We are disposed to give credence to this high praise bestowed upon it by Dr. Dixon, himself a distinguished oculist and a thor.

We have been particularly interested in the account given of the various eutozoa which infest the human eye and its appendages. Very handsome illustrations are given of the cysticercus cellulosæ, while full descriptions are afforded of the other hydatids of this locality.

The volume bears throughout the impress of that thorough scholarship which characterizes the Scotch as a people. The mere fact of its being a Scotch production is an argument in its favor, as we believe that race publishes as few useless books as any other.

